# Gynaecological Malignancies Among a Representative Population of Batticaloa, Sri Lanka

**DOI:** 10.7759/cureus.12947

**Published:** 2021-01-27

**Authors:** Markandu Thirukumar, Ahilan Sinnathurai

**Affiliations:** 1 Clinical Science, Faculty of Health Care Sciences, Eastern University, Batticaloa, LKA; 2 Pathology, Teaching Hospital, Batticaloa, LKA

**Keywords:** gynaecological malignancies, cervical cancer, corpus uteri cancer, histopathological subtypes, batticaloa, sri lanka

## Abstract

Background

Genital tract malignancies have a significant contribution to morbidity and mortality, particularly in resource-poor countries, including Sri Lanka. The distribution of such tumours varies from region to region.

Methodology

This was a retrospective, observational study at the Teaching Hospital, Batticaloa for five and a half years, from January 2012 to June 2017, and aimed at analyzing the pattern of gynaecological malignancies. All the histologically confirmed gynaecological cancers arising from the uterine cervix, endometrium, ovary, vagina, and vulva were included in the analysis.

Results

There were 508 cervical specimens to study histopathology of the cervix, 1,884 gynaecological specimens to study the endometrial histopathology, 537 ovarian specimens, and 92 vaginal and vulval specimen were sent for their histopathological study during the same period. About 143 genital tract malignancies had been diagnosed. There were 52 cervical malignancies (36.36%) and 52 ovarian malignancies (36.36%). The second commonest (20.28%) was endometrial malignancy. Vaginal malignancy was at fourth place (4.9%). Vulval malignancy was 2.1%. The peak age distribution of malignancies (55.24%) was mainly in the 40-59 years age range. The incidence of cervical and ovarian malignancies peaked at 40-59 years, with 32/52 (61.54 %) and 26/52 (50%) of the diagnosed cases, respectively*.*

Conclusion

Cervical cancer and ovarian cancer accounted for almost 72.73% of the entire gynaecological malignancies in this study, and both of them have the same peak incidence in the 40-59 age group. This study also showed that 43.36% of total female genital tract tumours are Human Papilloma Virus-associated cancers. They are not only preventable by certain strategies but also identifiable and manageable at the precancerous stage.

## Introduction

Genital tract malignancies have a significant contribution to morbidity and mortality, particularly in resource-poor countries, including Sri Lanka, where poor awareness and late presentation also prevail. Globally, gynaecological malignancies account for 10% of new malignancy cases in women and 12% of cancer deaths [1]. Most of the gynaecological malignancies differ in their frequency and patterns from one region to another. These wide variations in incidence could be because of the health-seeking behaviour of the women and services for organized screening for cervical malignancies, which is the commonest gynaecological malignancy in women worldwide [2,3,4]. In Sri Lanka, Human papillomavirus vaccination was introduced into the national vaccination programme in 2017. Two doses of vaccination are given at a six-month interval to students on completion of 10 years of age. The benefit of this primary preventive strategy is yet to be seen.

Squamous cell cervical carcinoma is the commonest female genital tract cancer in sub-Saharan Africa. Endometrial carcinoma is the commonest female genital tract cancers in North America and Northern Europe. The incidence of ovarian cancer has considerable geographical variation. Vulval and vaginal malignancies are rare and their highest incidence in the sixth decade of life [5].

In Sri Lanka, the incidence (crude rate) of cervix uteri and ovarian malignancies in 2014 were 9.8 and 8.4 per 100,000, respectively. They are the third and fourth commonest cancers in Sri Lanka. Breast and thyroid carcinoma were the first and second commonest during the same period. The incidence of uterine carcinoma was 5.4/100,000 population and was at the seventh place [6].

Sri Lanka is an island in the Indian Ocean with 21 million habitants that live in a land area of approximately 62,700 km2. The adult literacy rate is 89.7% for females and 92.6% for males. We conducted this study at the Teaching Hospital, Batticaloa, which is in the Eastern Province of Sri Lanka. 

This was a hospital-based study. As this is the only tertiary hospital with better health care facilities in this region, approximately 100% of the malignancies are treated here. Further, the study population included most of the diagnosed women with cancer from this region and therefore it could reflect the characteristics of the Batticaloa population; only a minority of women living in the region sought care outside this region. Therefore, this study reflects the gynaecological malignancies in this region despite it being a hospital-based study.

## Materials and methods

This was a retrospective, observational study at the Teaching Hospital, Batticaloa. This study included all patients that were diagnosed with gynaecological cancer over five and a half years, from January 2012 to June 2017, and aimed to analyze the pattern of gynaecological malignancies. All the histologically confirmed gynaecological cancers arising from the uterine cervix, endometrium, ovary, vagina, and vulva were included in the analysis. We collected the case records of all relevant patients with confirmed gynaecological cancer from medical records. They included the following details: age of patients, site of the tumour, and histopathological diagnosis.

The ethical review committee of the Faculty of Health Care Sciences, Eastern University, Sri Lanka issued this research approval - (EUSL/FHCS/ERC/2017/21). We processed data using SPSS version 21 (IBM Corp., Armonk, NY, USA). Descriptive statistics methods were used to analyze the results as whole numbers, percentages, tables, and charts.

## Results

There were 508 cervical specimens taken to study the histopathology of the cervix. It composed of a cervical biopsy from a punch biopsy or cone biopsy and cervical polypectomy. The specimens of endometrium were taken by endosampling, dilatation, and curettage for the evaluation of several symptoms and signs, such as abnormal uterine bleeding, lower abdominal pain, per vaginal bloody or brownish discharge. There were 1,884 gynaecological specimens to study endometrial histopathology. Among them, 1,642 specimens were adequate for a comprehensive diagnosis, and the remaining 242 (12.8%) were inadequate for reporting. About 242 (12.8%) of endometrial specimens were inadequate for a comprehensive diagnosis. We studied the histopathological pattern of 537 ovarian specimens. They were ovarian cysts from cystectomy, oophorectomy, salpingo-oophorectomy, and part of total abdominal hysterectomy (TAH). Ninety-two vaginal and vulval specimen were sent for their histopathological study during the same period.

There were 143 genital tract malignancies diagnosed from a total of 3,021 specimens for histopathology report, and we used all the cases for analysis. There were 52 cervical malignancies and 52 ovarian malignancies. Endometrial malignancy was the second commonest. Vaginal malignancy was at fourth place (Table 1).

**Table 1:** Frequency distribution of the gynaecological malignancies by sites

Site of tumour	Frequency (N=143)	Percentage (%) (100)
Cervix	52	36.36
Ovary	52	36.36
Endometrium	29	20.28
Vagina	7	4.90
Vulva	3	2.10

The peak age of occurrence of gynaecological malignancies was in the age range of 40-59 years with 79 patients (55.24%), followed by 60-69 years with 41 cases (28.67%), and 20-39 years with 16 cases (11.19%). The incidence of malignancies was 0.70% in the <19 years age group and 4.20% in the 70-79 years age group.

The distribution of the various gynaecological malignancies by age is also shown in Table 2. Majority of the cases of cervical malignancy were diagnosed between 40-59 years, with 32 out of 52 cases (61.54%) occurring in the fourth decade of life. Ovarian malignancy had a peak incidence in the age range of 40-59 years. 20 out of the 29 cases (68.97%) of endometrial cancer were diagnosed in the 40-59 years age range. The peak age of incidence of vaginal cancer was in the age range of 60-69 years, where five out of seven cases (71.42%) were found. Only three cases of vulval cancer were documented in the study period and they all were from the 60-69-year age group.

**Table 2:** Age distribution of gynaecological malignancies

Age	Cervical (52)	Ovarian (52)	Endometrial (29)	Vaginal (7)	Vulva (3)	Total (143)	Percentage (%)
<19	-	1	-	-	-	1	0.70
20-39	4	10	2	-	-	16	11.19
40-59	32	26	20	1	-	79	55.24
60-69	11	15	7	5	3	41	28.67
70-79	5	-	-	1	-	6	4.20

Table 3 shows that the main histological type of cervical cancer was squamous cell carcinoma, observed in 45 (84.6%) patients, while six (11.5%) patients showed adenocarcinoma. Adenocarcinoma was the only histological type of endometrial cancer seen, with 29 patients (100%). Similarly, all three vulval malignancies were squamous cell carcinoma type (100%). Most vaginal carcinoma (71.42%) were squamous cell carcinoma. Interestingly, one very rare type of vaginal cancer, malignant melanoma, was also reported during this study period. Among ovarian cancers, epithelial tumours were observed in 41 (78.85%) patients, followed by germ cell tumours in six (11.54%) patients, and sex cord tumour in five (9.61%) patients. 

**Table 3:** Site and histological pattern of gynaecological malignancies

Histological Type	Frequency	Percentage
Cervix		
Squamous cell carcinoma	45	84.6%
Adenocarcinoma	6	11.5%
Infrequent tumour subtypes	1	1.9%
Total	52	100%
Corpus Uteri		
Adenocarcinoma	29	100%
Vulva		
Squamous cell carcinoma	3	100%
Vaginal carcinoma		
Squamous cell carcinoma	5	71.42%
Malignant melanoma	1	14.29%
Lympho-epithelioma like carcinoma	1	14.29%
Total	7	100%
Ovarian		
Epithelial tumour		78.85%
Serous cystadenocarcinoma	31	
Mucinous cystadenocarcinoma	5	
Clear cell carcinoma	5	
Germ cell tumour		11.54%
Dysgerminoma	5	
Struma ovarii	1	
Sex cord stromal tumour		9.61%
Granulosa cell tumour	5	
Total	52	100%

Serous adenocarcinoma was the most common histological type of epithelial ovarian cancer with 31 patients, and struma ovarii was the least, occurring only in one patient. All other tumours such as mucinous cystadenocarcinoma, clear cell carcinoma, dysgerminoma, and granulosa tumour were distributed equally.

## Discussion

There were 18.1 million new cancer cases and 9.6 million cancer deaths worldwide in 2018, and nearly half of them were in Asia, as this region has nearly 60% of the global population [7].

The study showed cervical malignancies contributed 36% of the genital tract malignancies. It was lower than the outcome of the study by Jamal et al. in Pakistan, where cervical malignancies accounting for 47% of all gynaecological cancers [8]; 61.5% from Sokoto and 63.1% from Port-Harcourt [9,10]; and studies in Enugu and Maiduguri, where 78% and 70.5% were documented, respectively [11,1]. 

In the present study, cervical and ovarian malignancies were the commonest gynaecological malignancies accounting 36.36% in each, followed by uterine (20.28%), vaginal (4.9%), and vulval (2.1%).

The incidence of cervical and ovarian malignancies were 61.9% and 23.9%, respectively, in a study by Madhutandra et al. [12]. The incidence of these tumours was 65.26% and 22.11% in a study by Chaudhary et al. [13]. While the incidence of cervical carcinoma and ovarian carcinomas were 86.1% and 4.68%, respectively, in a study by Jhansivani et al. [14]. The study of Nasreen [15] reported ovarian cancer more than cervical cancer (48%, 24% respectively). Similarly, ovarian cancer was higher than cervical cancer in a study conducted in Tehran: 55.5% of former and 19.6% of the latter [16].

In Sri Lanka, cervical cancer was the third most common female cancer in 2014 and contributed 8.2% of all female cancers. Ovarian cancer, the fourth most common female cancer, was 7.2% of all female cancers [6]. There was no well-established population-based cancer registry in Sri Lanka. Most of the data are from hospital- and laboratory-based active collection. Therefore, countrywide true data was not available for comparison. In addition, delayed health-seeking behaviour of the population was also common in developing countries, including Sri Lanka. These factors could be the reasons for the underestimation of the true incidence of the cancers.

Further, lesser incidence of teenage sexual activities in the studied region and improved coverage of Pap smear programme for early detection of premalignant dysplasia might have contributed in a less proportion of cervical malignancies in Batticaloa when compared with other developing countries.

The age range of the study population ranged from 19 to 79 years, and the peak incidence of cancer (55.2%) was in the 40-59 years age range. The peak age range of incidence of cervical malignancies in this study was 40-59 years. This was consistent with the results of Yakasai et al., Briggs et al., and Jamal et al., who reported 46.25±4.99 years, 42 years, and 47 years, respectively. The lower incidence of teenage sexual activities in the studied region and improved coverage of the Pap smear programme for early detection of premalignant dysplasia might have contributed to a lower proportion of cervical malignancies in Batticaloa when compared with other developing countries [17,18,8].

This study was similar to studies done in the North-Eastern part of India, where the most common age group for carcinoma of the cervix was 51-60 years [19]. Another study in India also found that the most common age group for cancer cervix was 41.50 years (mean age = 49.53 years) [20]. Ovarian cancer was contributing to 36.6% of all gynaecological cancers in this study. It was higher than what was reported in Kano by Yakasai et al. (17), in Maiduguri by Kyari et al. [1], in Enugu by Ugwu et al. [11], and in Enugu by Okeke et al. [21]. The incidence of ovarian cancer in this study was at a peak in the age of 40-59 years. This is similar to what is found in the literature [22], where it has been documented that worldwide, the highest incidence was in the 40-49 years age group.

The incidence of gynaecological malignancies was least in the young and old populations. The natural history of cervical cancer takes over 10 years to transform into the asymptomatic stage when the patient presents to the hospital. It is, therefore, unlikely to present earlier than 20 years of age. Further, most of the elderly population that develops the disease, because of the late presentation, might have died before the age of 70 years. 

Overall, cervical and ovarian cancers accounted for almost 72.73% of the entire gynaecological malignancies in this study, and both of them had the same peak incidence in the 40-59 age group. The rest of the gynaecological cancers, including corpus uteri, vulva, and vaginal cancers accounted for the remaining 17.27%. Thus, cancer continues to claim the lives of women in developing countries when they are critical to social and economic stability.

This study showed that 43.36% of total female genital tract tumours, such as cancer of cervix, vulva, and vagina, are Human Papilloma Virus (HPV)-associated cancers. They are not only preventable by certain strategies but also identifiable and manageable at the premalignant stage. This can be achieved through strengthening the primary and secondary preventive strategies in developing countries such as Sri Lanka.

the incidence and age-standardized rates of endometrium cancer for the world were 4.8% and 8.3%, respectively; for India, they are 2.3% and 2.3%, respectively [23]. In the US, 1 in 40 women develops endometrial cancer in her lifetime [24]. The incidence was higher in the white population of the US and lowest in India and Japan [24, 25]. In India, endometrial cancer ranks third among genital malignancies, after cancers of cervix and ovary [24]. In our study also, we have found similar results. In a study in India, the most common age group for carcinoma of endometrium was 51-60 years, with a mean age of 53.76 years; though the same had been reported as 55-64 years, with a mean age of 61.0 years [25]. In this study also, we found that the peak incidence of endometrial cancer was in the 40-59 years age range.

Limitations of the study

This study shares the limitations expected in hospital-based secondary data research. Although this hospital is a Tertiary Care Teaching hospital, there may be patients who did not seek care from this hospital, such as those who trust private-sector Western health care or alternative medicine. Data collection was not from a tool primarily designed for this study and many variables may not be partly or fully covered. Since a paper-based health information system is used in this hospital, it is likely some patients may have been missed. There may a bias due to selective health-seeking by the high-risk population.

## Conclusions

Primary prevention is better and less expensive than tertiary prevention. The HPV-inflicted malignancies, such as cervical, vulval, and vaginal carcinomas, are preventable by certain strategies and identifiable at their pre-malignant stages by participating in a cancer-screening programme. Public health education aimed to establish personal abstinence from sex, avoiding sexual promiscuous behaviours, and using condoms in a high-risk sexual relationship could be commenced during teenage. In addition, the public could be encouraged for HPV vaccination for their school-going children. Policymakers should be mindful in improving the socio-economic status of the people for better living conditions, which can also decrease cancer incidence.

There are no recommended screening tests for ovarian cancer for women who do not have symptoms and are not at high risk of developing ovarian cancer. However, transvaginal ultrasound and CA-125 may be useful to screen women who have a high risk of ovarian cancer. Regarding ovarian cancer, improvement in awareness of the symptoms of ovarian cancer is helpful in diagnosing the condition at an early stage. General practitioners and women need to know that women with ovarian cancer do experience symptoms, even though they are non-specific, more frequently, severely, and persistently than those without the disease. Seeking medical advice early without delay when these symptoms develop is vital for a better prognosis.
